# Video-assisted retroperitoneal debridement for infected retroperitoneal fat necrosis following laparoscopic distal pancreatectomy

**DOI:** 10.1007/s12328-026-02414-0

**Published:** 2026-08-01

**Authors:** Katsuya Ami, Keiko Kamei, Masaya Nakano, Chihoko Nobori, Yuta Yoshida, Kentaro Tai, Takaaki Murase, Atsushi Takebe, Ippei Matsumoto

**Affiliations:** Division of Hepato-Biliary-Pancreatic Surgery, Department of Surgery, Faculty of Medicine, Kindai University, 1-14-1 Mihara-Dai, Minami-Ku, Sakai, Osaka 590-0197 Japan

**Keywords:** Laparoscopic distal pancreatectomy, Retroperitoneal fat necrosis, Step-up approach, Video-assisted retroperitoneal debridement

## Abstract

Video-assisted retroperitoneal debridement (VARD) is an established and minimally invasive treatment option for infected necrotizing pancreatitis. However, its application in the postoperative setting after pancreatic resection has rarely been described. We report a case of extensive infected retroperitoneal fat necrosis following laparoscopic distal pancreatectomy that was successfully managed using a step-up approach incorporating VARD. An 81-year-old woman underwent laparoscopic distal pancreatectomy for pancreatic ductal adenocarcinoma of the pancreatic tail. On postoperative day (POD) 7, she developed sudden-onset abdominal pain. Computed tomography (CT) demonstrated enlargement of the remnant pancreas with increased peripancreatic fat stranding. Follow-up CT on POD 9 and 12 revealed progressive enlargement of the fluid collection around the pancreatic stump with rapid extension of inflammatory changes into the retroperitoneal space. Conservative management with fasting and antibiotic therapy resulted in temporary improvement in inflammatory markers. However, extensive infected retroperitoneal fat necrosis developed on POD 38. On POD 43, CT-guided percutaneous catheter drainage failed to achieve adequate source control due to highly viscous necrotic debris, necessitating surgical necrosectomy. An additional drainage tube was placed to establish a retroperitoneal access route, after which VARD was performed on POD 55, with removal of 61 g of necrotic fatty tissue. Inflammatory markers rapidly improved, and the patient was discharged in good condition on POD 102. Infected retroperitoneal fat necrosis after laparoscopic distal pancreatectomy was successfully managed using a step-up strategy incorporating VARD. This case highlights VARD as a minimally invasive therapeutic option for postoperative infected retroperitoneal fat necrosis.

**Supplementary Information:**

The online version contains supplementary material available at https://doi.org/10.1007/s12328-026-02414-0.

## Introduction

Video-assisted retroperitoneal debridement (VARD) is an established highly effective minimally invasive treatment option for infected necrotizing pancreatitis [1, 2]. VARD is typically performed as part of a step-up approach when the initial drainage, such as percutaneous catheter drainage (PCD) or endoscopic intervention, fails to achieve adequate source control [3]. By inserting a videoscope through a small incision to perform necrosectomy, VARD allows the safe removal of deep necrotic tissue that is difficult to visualize under direct vision, and has been reported to reduce invasiveness and improve clinical outcomes compared with conventional open necrosectomy [3–5].

Herein, we report a case of extensive infected retroperitoneal fat necrosis following laparoscopic distal pancreatectomy. The condition was refractory to conservative management and standard drainage but was successfully managed using a therapeutic strategy incorporating VARD.

### Case report

**Fig. 1:**
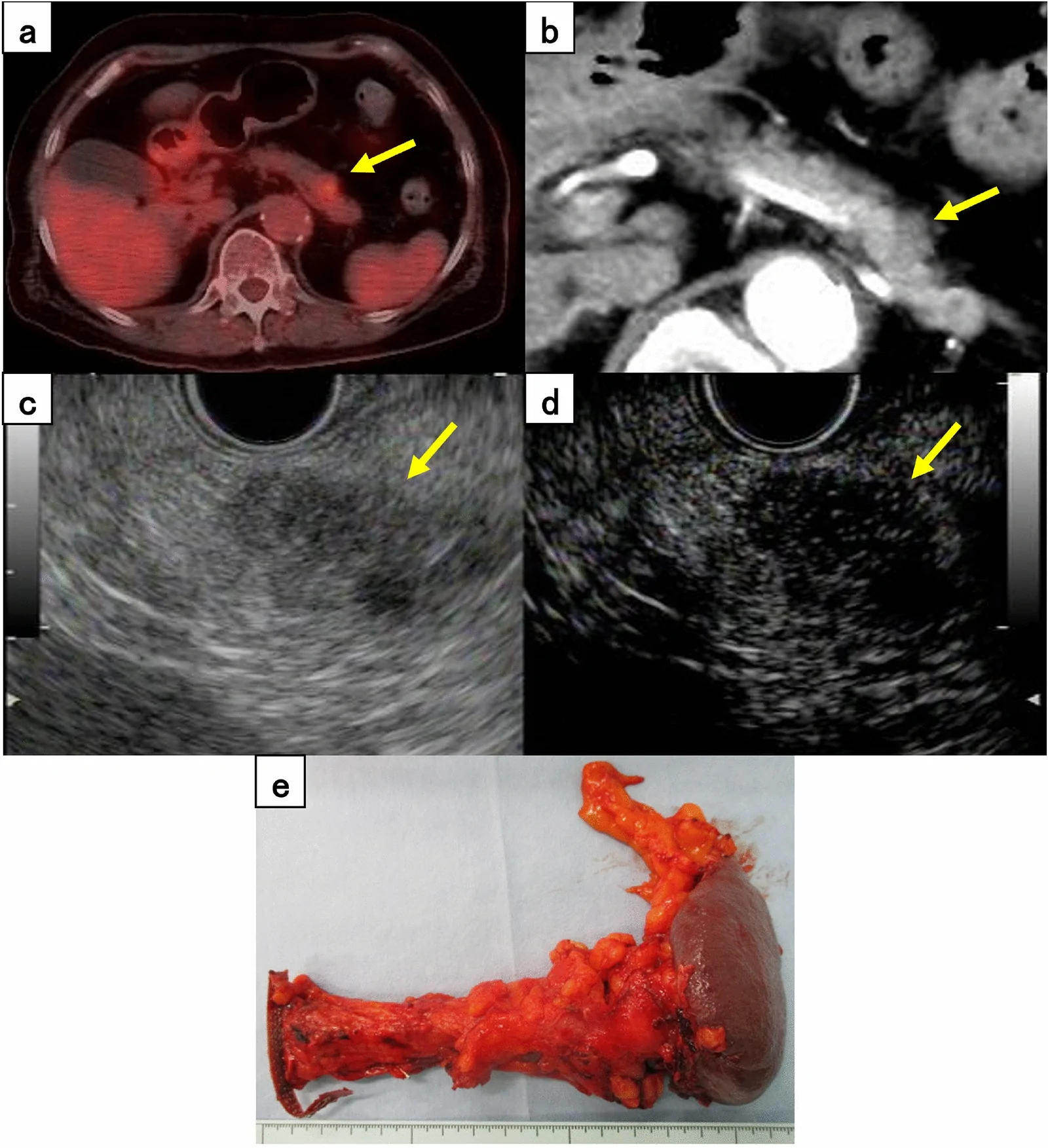
Positron emission tomography-computed tomography (CT) shows focal uptake in the pancreatic tail (arrow) **a**. Contrast-enhanced CT demonstrates a small hypovascular mass in the pancreatic tail with distal pancreatic duct dilatation and parenchymal atrophy (arrow) **b**. Contrast-enhanced harmonic endoscopic ultrasonography using perflubutane microbubbles (Sonazoid®, GE Healthcare) shows a hypovascular hypoechoic lesion in the pancreatic tail (arrow) **c**, **d**. Resected specimen after distal pancreatectomy and splenectomy performed via a laparoscopic approach **e**

An 81-year-old woman with diffuse large B-cell lymphoma (DLBCL) who achieved remission after R-THP-COP (rituximab, pirarubicin, cyclophosphamide, vincristine, and prednisolone) chemotherapy was referred to our institution after incidental uptake in the pancreatic tail was detected on surveillance positron emission tomography-computed tomography (CT) (Fig. 1a). Her medical history included hypertension and diabetes mellitus. Physical examination was unremarkable. Laboratory tests revealed hemoglobin A1c level of 7.1%, whereas other laboratory parameters were within normal limits. Tumor markers revealed mildly elevated carcinoembryonic antigen level (5.8 ng/mL) and normal carbohydrate antigen 19-9 levels. Contrast-enhanced CT revealed a 13-mm hypovascular mass in the pancreatic tail, with distal pancreatic duct dilatation and parenchymal atrophy without regional lymphadenopathy, distant metastasis, or recurrence of DLBCL (Fig. 1b). Endoscopic ultrasonography (EUS) revealed a hypoechoic lesion in the pancreatic tail that appeared hypovascular on contrast-enhanced harmonic EUS using perflubutane microbubbles (Sonazoid®, GE Healthcare) (Fig. 1c, d). EUS-guided fine-needle aspiration confirmed the diagnosis of adenocarcinoma. Based on these findings, the patient was diagnosed with resectable pancreatic tail cancer and underwent a laparoscopic distal pancreatectomy with curative intent. The procedure was performed using a radical antegrade modular pancreatosplenectomy (RAMPS) approach with dissection along the fusion fascia of Toldt and partial resection of the Gerota fascia near the tumor. The pancreas was soft, and transection at the left border of the portal vein was completed using a 60-mm reinforced linear stapler (Endo GIA™ Reinforced Reload with Tri-Staple™ Technology, Medtronic, Minneapolis, MN, USA). A closed drain was placed adjacent to the pancreatic stump (operative time: 324 min; blood loss: 200 mL) (Fig. 1e). Perioperative antibiotic prophylaxis with cefmetazole was administered on the day of surgery and continued until postoperative day (POD) 2. Histopathological examination revealed a well-differentiated invasive pancreatic ductal adenocarcinoma (pT1, pN1, cM0, Stage IIB) according to the UICC 9th edition [6].

**Fig. 2:**
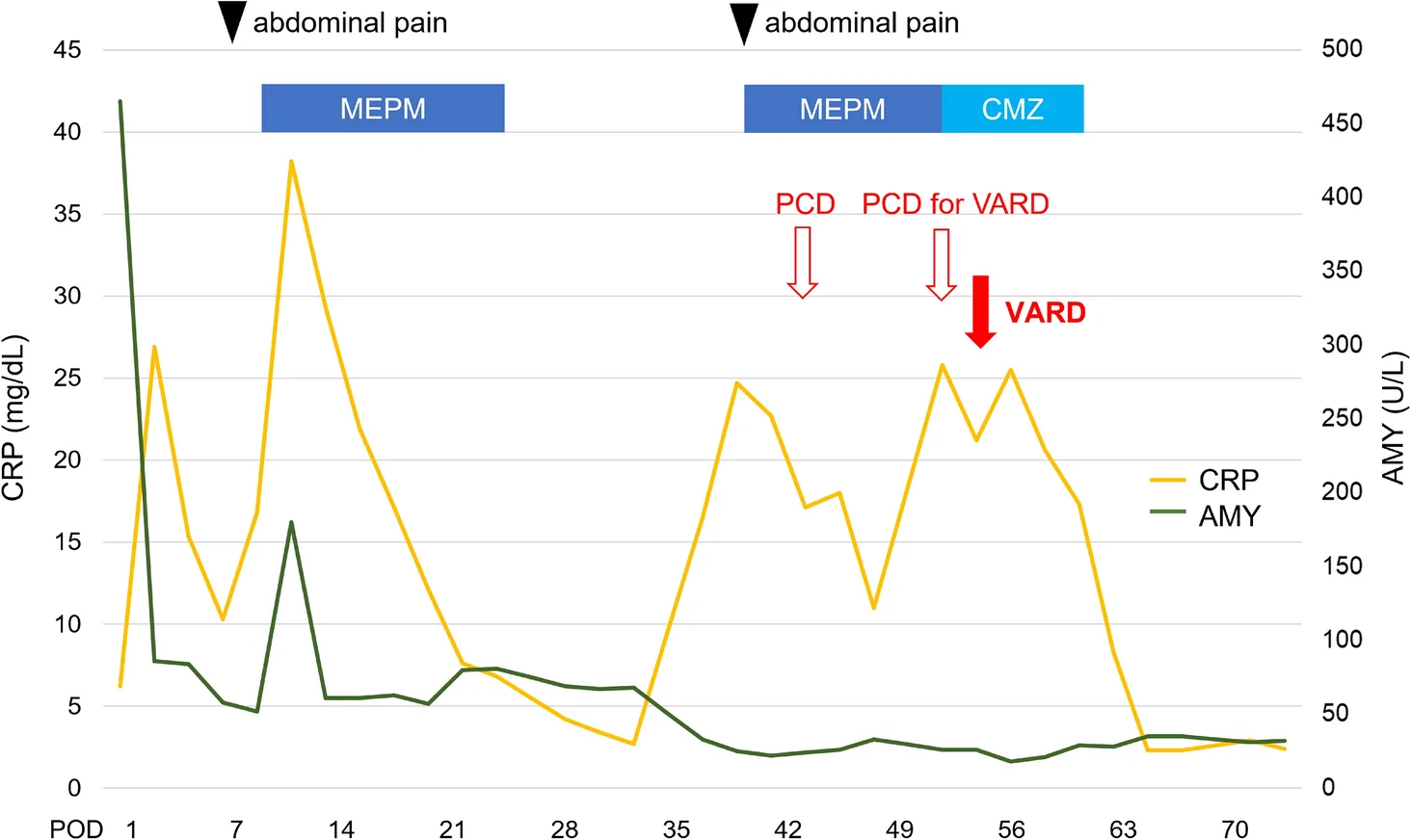
Postoperative clinical course and therapeutic interventions. Serum C-reactive protein levels and serum amylase levels are shown during the postoperative period. The timing of antibiotic therapy, percutaneous catheter drainage, and video-assisted retroperitoneal debridement is indicated. AMY: amylase, CMZ: cefmetazole, CRP: C-reactive protein, MEPM: meropenem, PCD: percutaneous catheter drainage, VARD: video-assisted retroperitoneal debridement

**Fig. 3:**
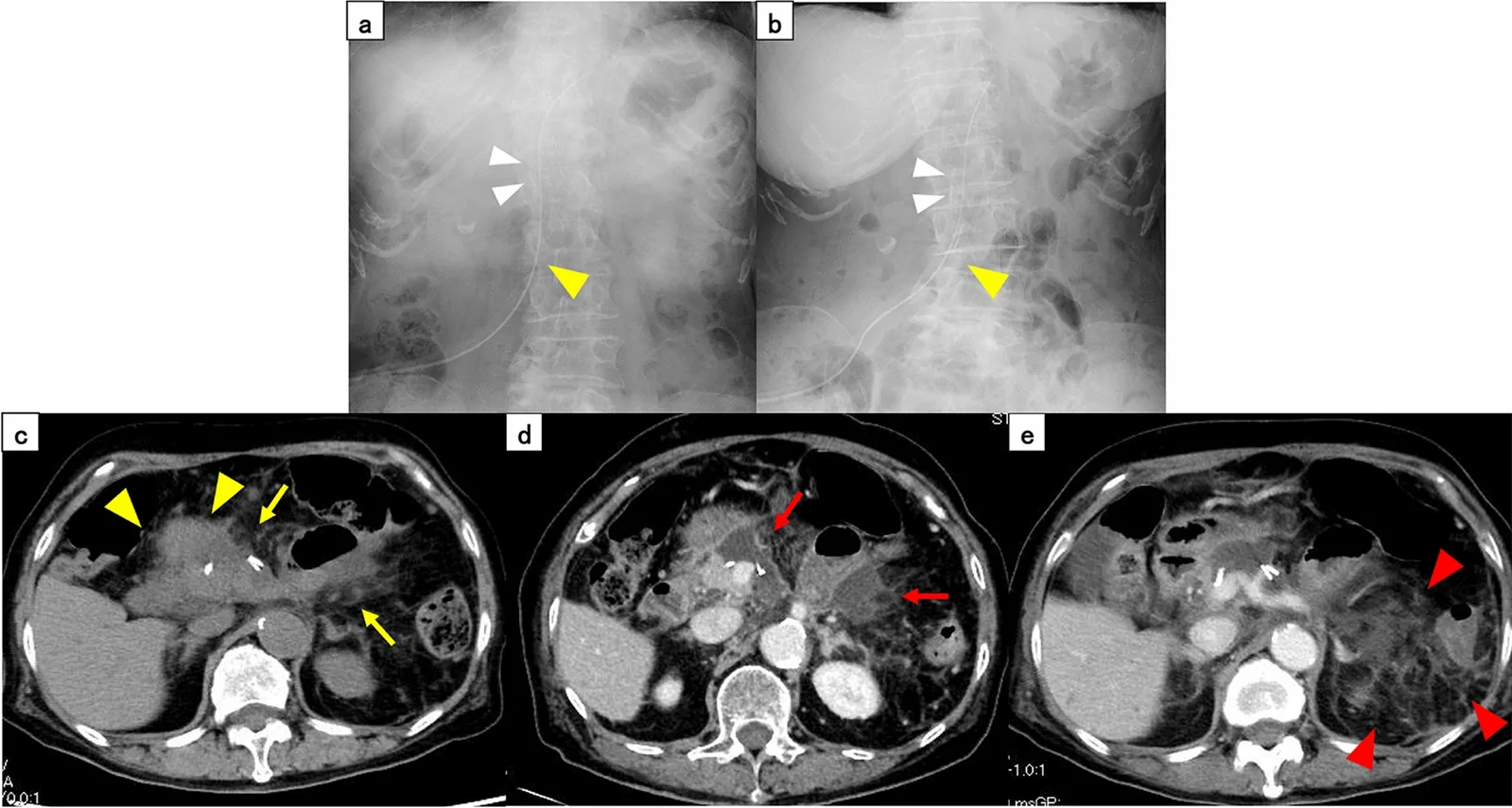
Postoperative day (POD) 1 and 3: Abdominal radiographs show a closed-suction drain (yellow arrowhead) placed adjacent to the pancreatic stump (white arrowhead) **a**, **b**. POD 7: Computed tomography (CT) shows enlargement of the remnant pancreas (yellow arrowhead) with surrounding fat stranding (yellow arrow) **c**. POD 9: Contrast-enhanced CT shows enlargement of the fluid collection (red arrow) and progression of peripancreatic inflammatory changes **d**. POD 12: Contrast-enhanced CT shows inflammatory changes extending into the left retroperitoneal space (red arrowhead), without pancreatic parenchymal necrosis or loss of enhancement **e**

**Fig. 4:**
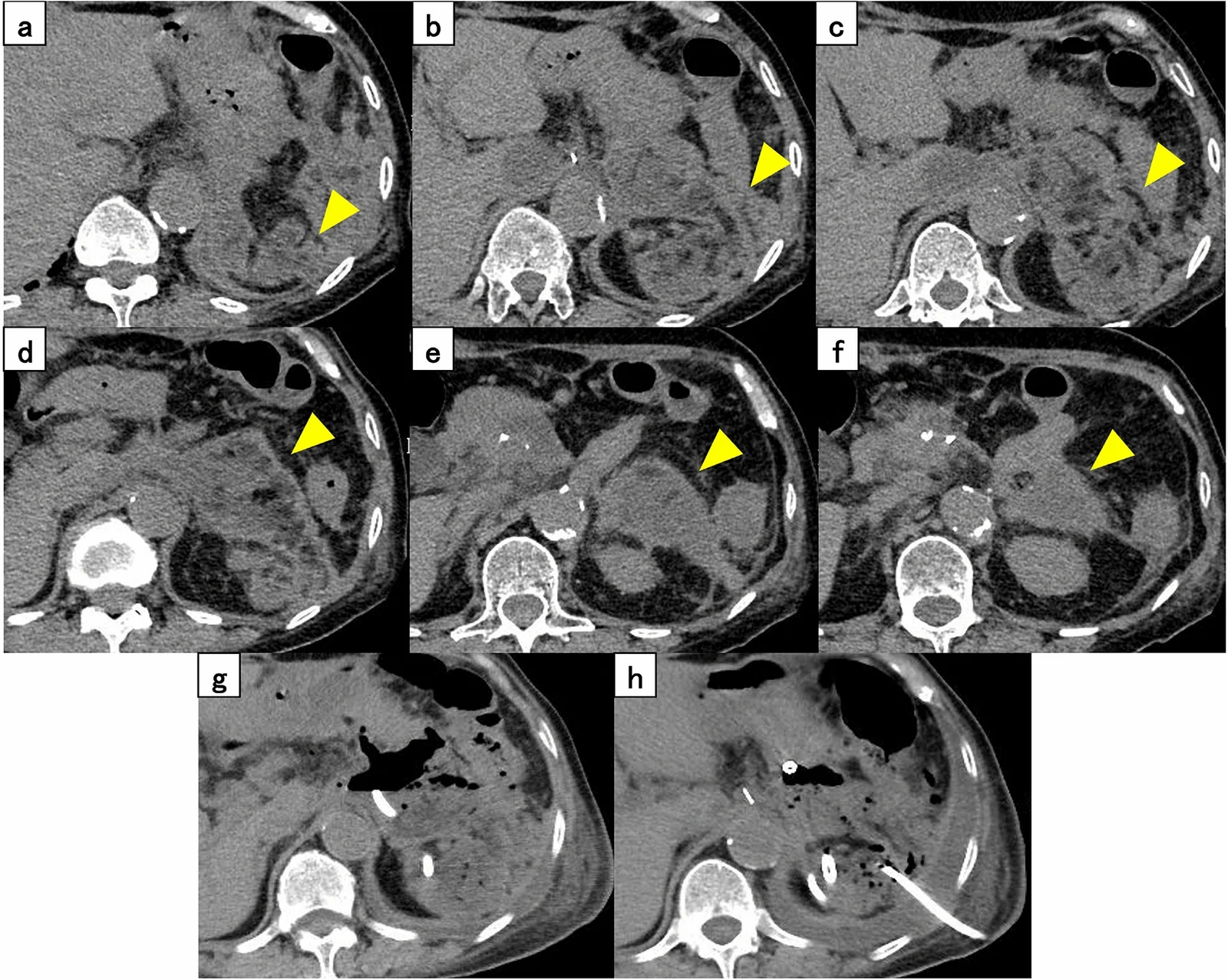
Sequential axial computed tomography (CT) images obtained at the same examination demonstrate a large heterogeneous low-attenuation area with internal fluid components and marked fat stranding widely extending throughout the left retroperitoneal space **a**–**f**. CT-guided percutaneous catheter drainage demonstrating placement of drainage tubes within the necrotic cavity **g**, **h**

**Fig. 5:**
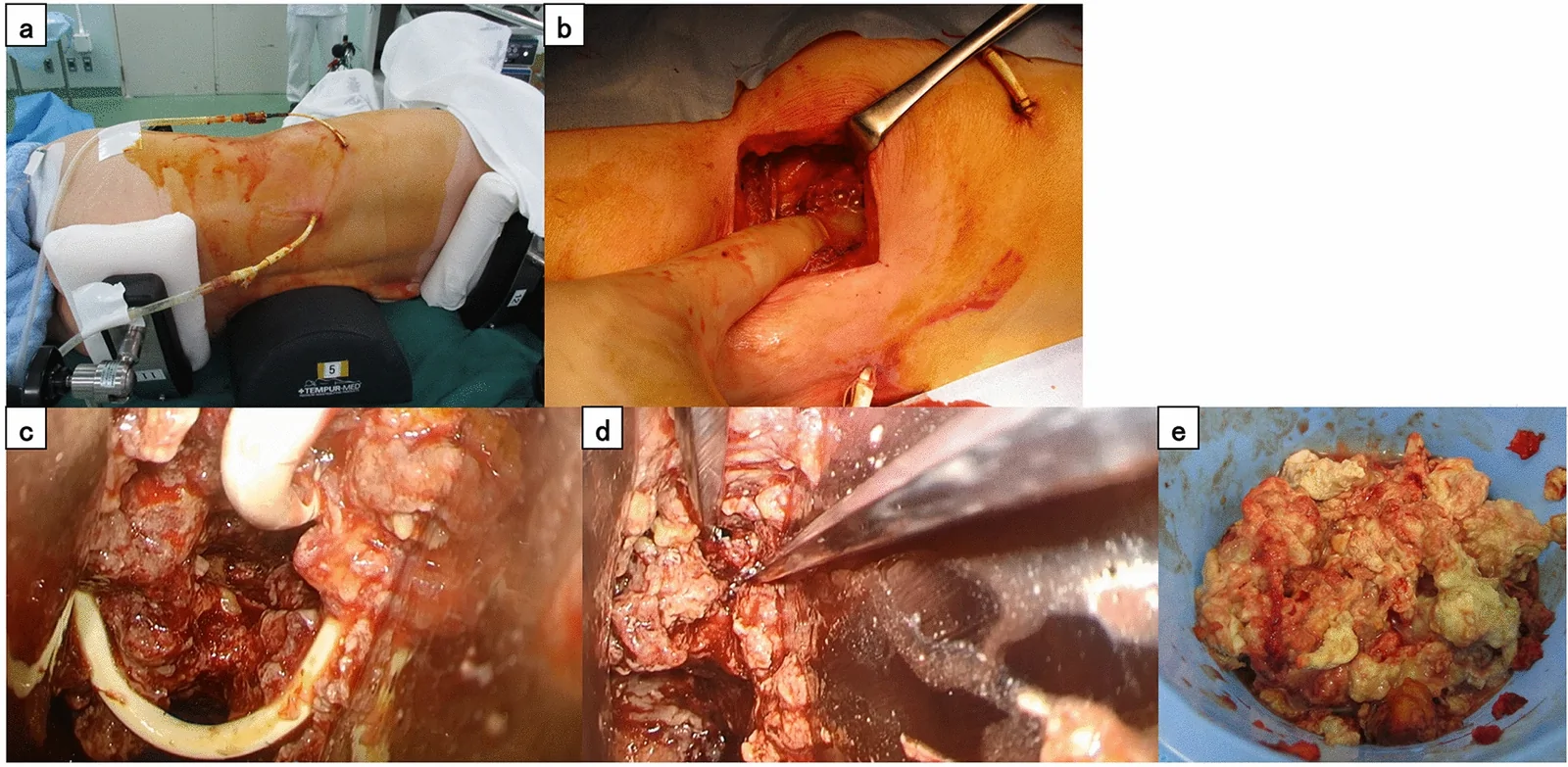
The patient was placed in the right semi-lateral decubitus position **a**. A 6-cm incision was made below the left costal margin between the existing drainage tracts **b**. The necrotic cavity was identified and opened along the drainage tract. Necrotic fatty tissue was debrided under endoscopic visualization **c**, **d**. Resected necrotic fatty tissue weighing 61 g **e**

The postoperative course is summarized in Fig. 2. Serum amylase levels on PODs 1 and 3 were 465 U/L and 86 U/L, respectively. Abdominal radiographs obtained on PODs 1 and 3 confirmed that the drain remained positioned adjacent to the pancreatic stump (Fig. 3a, b). Amylase levels in the drain fluid were 1,632 U/L and 372 U/L on PODs 1 and 3, respectively. The drain output, consisting of a thin serosanguineous fluid, was 100 mL on POD 1 and 36 mL on POD 3. The drain was removed on POD 4. Drain fluid culture was not performed before removal because there were no clinical signs of infection. On POD 7, the patient suddenly developed abdominal pain with elevated inflammatory markers (C-reactive protein [CRP] 10.3 mg/dL, white blood cell count [WBC] 10,336 /µL). Serum amylase and lipase levels were within the normal range (64 U/L and 47.9 U/L, respectively). CT demonstrated enlargement of the remnant pancreas with surrounding fat stranding (Fig. 3c). A postoperative peripancreatic inflammatory process, possibly related to insufficient drainage, was suspected, and the patient was initially managed with close observation. On POD 9, the abdominal pain worsened and the inflammatory markers markedly increased (CRP 26.7 mg/dL, WBC count 25,054 /µL). Repeat contrast-enhanced CT demonstrated enlargement of the fluid collection around the pancreatic stump, with rapidly progressive peripancreatic inflammatory changes extending toward the retroperitoneal space (Fig. 3d). A postoperative inflammatory process with retroperitoneal extension was considered. Given the rapidly progressive retroperitoneal inflammatory changes and the lack of available microbiological data, blood cultures were obtained, and empirical broad-spectrum antimicrobial therapy with meropenem was initiated. The blood cultures subsequently remained negative. Further contrast-enhanced CT on POD 12 demonstrated the progression of retroperitoneal inflammation without pancreatic parenchymal necrosis or loss of enhancement (Fig. 3e). The inflammatory markers and abdominal symptoms gradually improved with conservative management. However, on POD 38, the patient developed a fever, abdominal pain, and recurrent elevation of inflammatory marker levels. The CT findings were consistent with infected retroperitoneal fat necrosis demonstrating a large heterogeneous low-attenuation area with internal fluid components and marked fat stranding that extended widely throughout the left retroperitoneal space (Fig. 4a–f). Therefore, meropenem antibiotic therapy was initiated. Although the inflammatory markers showed partial improvement, they did not normalize, and the necrotic cavity size did not decrease. CT-guided PCD was performed on POD 43 (Fig. 4g). Cultures of the drainage fluid grew extended-spectrum β-lactamase-producing *Escherichia coli* and *Enterococcus* species. Antibiotic therapy was de-escalated to cefmetazole based on the results of antimicrobial susceptibility testing. No significant drainage was obtained and adequate source control could not be achieved. Surgical necrosectomy was considered necessary and VARD was planned as a minimally invasive approach. An additional CT-guided drainage tube was placed on POD 53 to establish an optimal access route for the VARD (Fig. 4h), which was subsequently performed on POD 55 (Fig. 5a–e). The details of the VARD procedure are provided in Supplementary file 1: Video S1. The patient was placed in the right semi-lateral decubitus position, and a 6-cm incision was made below the left costal margin between the existing PCD tracts. After dividing the subcutaneous tissue and latissimus dorsi muscle, the necrotic cavity was identified by digital palpation along the drainage tract and opened. Under endoscopic visualization, 61 g of necrotic fatty tissue was debrided and removed (operative time, 57 min; minimal blood loss). The inflammatory marker levels improved, along with the patient’s general condition. The residual retroperitoneal abscess gradually resolved, and the drain was exchanged and subsequently removed on POD 85. The patient continued rehabilitation and was discharged in good condition on POD 102. However, adjuvant chemotherapy was not administered because of the patient’s advanced age and decline in physical condition following the prolonged postoperative course associated with severe infectious complications. Lymph node recurrence involving the left supraclavicular and para-aortic lymph nodes developed 15 months after surgery, and S-1 chemotherapy was initiated as treatment for recurrent disease. The patient succumbed to the disease 52 months after the initial procedure.

## Discussion

In the present case, extensive infected retroperitoneal fat necrosis developed after laparoscopic distal pancreatectomy and was successfully managed using a step-up approach incorporating VARD. VARD is a minimally invasive surgical technique used for the management of infected necrotizing pancreatitis and was successfully adapted for this postoperative complication [1, 7]. Infected retroperitoneal fat necrosis following pancreatic resection has rarely been reported, and, to the best of our knowledge, the successful management of this condition using VARD has not been previously reported. This case highlights VARD as a promising therapeutic option for postoperative infected retroperitoneal fat necrosis.

Peripancreatic inflammation can extend into the retroperitoneal space and progress to infected necrosis [8–10]. In the present case, the inflammatory changes initially improved with conservative management without the need for additional drainage, but later progressed to delayed infected retroperitoneal fat necrosis, requiring further intervention. The pathophysiology of this case was likely multifactorial. As enlargement of the fluid collection around the pancreatic stump and localized inflammatory changes developed after drain removal, minor pancreatic leakage or insufficient postoperative drainage could not be excluded. In addition, the onset on POD 7 was later than the current temporal definition of post-pancreatectomy acute pancreatitis (PPAP) [11], and the elevation in pancreatic enzyme levels was mild, suggesting delayed postoperative peripancreatic inflammation rather than the typical early PPAP [12]. Although the initial episode responded to conservative treatment, subclinical inflammation or residual necrotic changes may have persisted and subsequently became clinically overt, resulting in infected retroperitoneal fat necrosis. Fat necrosis involving the peripancreatic and retroperitoneal regions is a known pathological finding in acute pancreatitis. However, delayed clinically meaningful infected retroperitoneal fat necrosis requiring surgical intervention after pancreatic resection is uncommon [13, 14]. In addition, advanced age, diabetes mellitus, and a history of chemotherapy for DLBCL may have contributed to prolonged infection through impaired host defense and increased susceptibility to infection. Furthermore, retroperitoneal dissection and partial resection of the Gerota fascia associated with RAMPS may have contributed to postoperative anatomical changes and exposure of the retroperitoneal fat tissue, thereby facilitating retroperitoneal extension of the inflammatory process [15]. However, no clear evidence supports the association between RAMPS and retroperitoneal fat necrosis, and causality cannot be established from this single case.

Although specific treatment guidelines for infected retroperitoneal fat necrosis have not been established, management strategies for the analogous condition of necrotizing pancreatitis are well defined. Traditionally, necrotizing pancreatitis was managed using open drainage and necrosectomy [16, 17]; however, these procedures are highly invasive and are associated with substantial morbidity and mortality. In recent years, a step-up approach consisting of an initial percutaneous or endoscopic drainage followed, when necessary, by minimally invasive necrosectomy has been recommended. Randomized controlled trials have demonstrated that this strategy significantly reduces the rate of major complications or mortality compared with open necrosectomy (40% vs. 69%) [3, 18]. Furthermore, endoscopic necrosectomy is currently regarded as a less invasive treatment option [19, 20]; however, a transgastric approach may not always be feasible depending on the location and extent of necrotic lesions. In such cases, a surgical approach that allows direct removal of necrotic tissue may be an effective therapeutic option. In the present case, broad-spectrum antibiotic therapy failed to achieve adequate infection control, and the extensive infected retroperitoneal fat necrosis was initially managed using CT-guided PCD. However, highly viscous necrotic fatty debris could not be adequately removed using catheter drainage alone, and additional debridement was deemed necessary. In accordance with the principles of the step-up approach, endoscopic drainage and/or necrosectomy were also considered. Nevertheless, necrotic lesions were widely distributed within the retroperitoneal space and predominantly involved the left dorsolumbar region, making it difficult to secure an adequate transgastric drainage route. Therefore, VARD was considered more suitable than endoscopic necrosectomy because it allowed direct retroperitoneal access and more effective debridement of the necrotic tissue. Although earlier intervention may be considered in retrospect, the patient initially responded to conservative treatment with improvement in both clinical symptoms and inflammatory markers, making surgical intervention difficult to justify at that stage. Only after delayed infected retroperitoneal fat necrosis became clinically evident and percutaneous drainage failed to achieve adequate source control did VARD become an appropriate therapeutic option. In the present case, prolonged severe postoperative infectious complications precluded the timely initiation of adjuvant chemotherapy, which may have adversely affected subsequent oncological management. Although VARD successfully achieved infection control in a minimally invasive manner, this case highlights the importance of preventing postoperative infectious complications and achieving timely source control to optimize oncological outcomes in patients with pancreatic cancer.

In VARD, a preoperatively placed PCD catheter is used to guide access to the necrotic cavity through a small skin incision via a retroperitoneal approach [2]. Necrotic debris in areas that are not readily visible under direct vision can be safely removed by introducing a videoscope through a limited incision and performing necrosectomy under endoscopic visualization. Compared with open necrosectomy, this technique is considered less invasive and is associated with reduced surgical trauma [3, 5]. In the present case, the initial PCD catheter was inserted through the left dorsolumbar musculature, resulting in inadequate access to the necrotic cavity within the retroperitoneal space. Therefore, a second drainage catheter was placed in the left 11th intercostal space. A 6-cm skin incision was created between the two drainage catheters, and the retroperitoneal space was accessed using both catheters as guides, enabling adequate debridement of the necrotic tissue. This case suggests that VARD can be used effectively to treat infected retroperitoneal fat necrosis that develops after pancreatic resection. In particular, when necrotic lesions are widely distributed within the retroperitoneal space and a transgastric approach is not feasible, VARD allows direct retroperitoneal access and may represent an anatomically rational therapeutic option.

In conclusion, infected retroperitoneal fat necrosis can occur as a postoperative complication after distal pancreatectomy and was successfully managed in the present case using a step-up strategy incorporating VARD. These findings suggest that VARD, an established treatment for necrosis associated with acute pancreatitis, may also serve as an effective and minimally invasive treatment for postoperative retroperitoneal fat necrosis. In particular, when necrotic lesions are widely distributed within the retroperitoneal space, and a transgastric approach is not feasible, VARD may be a useful therapeutic option.

## Supplementary Information

Below is the link to the electronic supplementary material.

Supplementary file1 (MP4 27606 kb)
